# Emotional problems mediate the association between attention deficit/hyperactivity disorder and obesity in adolescents

**DOI:** 10.1186/s12888-023-04882-x

**Published:** 2023-05-31

**Authors:** Ke Li, Liangliang Chen, Kai Wang, Xiaodong Jiang, Yiting Ji, Shuanfeng Fang, Haiyan Wei

**Affiliations:** 1 Department of Child Health Care, Children’s Hospital Affiliated to Zhengzhou University, Zhengzhou, China; 2 Department of Developmental and Behavioral Pediatrics, (Fujian Branch of Shanghai Children’s Medical Center), Fujian Children’s Hospital, Fuzhou, China; 3 Department of Pediatrics, 1st Affiliated Hospital to Zhengzhou University, Zhengzhou, China; 4 Rehabilitation Medicine Department, Wuhan Children’s Hospital(Wuhan Maternal and Child Healthcare Hospital), Tongji Medical College, Huazhong University of Science & Technology, Wuhan, China; 5 Department of Child and Adolescent Healthcare, Children’s Hospital of Soochow University, Suzhou, China; 6 Department of Endocrinology and Genetic Metabolism, Children’s Hospital Affiliated to Zhengzhou University, Zhengzhou, China

**Keywords:** ADHD, Body mass index, Anxiety, Depression, Adolescent, Obesity

## Abstract

**Background:**

Attention deficit/hyperactivity disorder (ADHD) has been identified as a risk factor for obesity in both children and adolescents. However, the mechanisms underlying the relationship between ADHD and obesity are still unclear. This study aimed to test a theoretical model of whether anxiety/depression is an intermediary factor in the ADHD-obesity relationship.

**Methods:**

Data were derived from the National Health Interview Survey (NHIS), a principal source of information on the health of the civilian noninstitutionalized population of the United States. A total of 35,108 adolescents aged 12-17 years old from 2010-2015 NHIS and 2016-2018 NHIS representing 46,550,729 individuals in the weighted population, had a parent-reported previous ADHD diagnosis, emotional problems, and height and weight data. Mediation analyses were used to explore whether anxiety/depression is an intermediary factor in the relationship between ever having ADHD and obesity. Mediation analyses were performed using multiple logistic regressions.

**Results:**

The findings showed that ADHD was a predictor of obesity. This relationship was partially mediated by depression(2010-2015: β=0.28, 95%CI:0.13-0.43; 2016-2018: β=0.26, 95%CI:0.03-0.49), as well as anxiety (2010-2015: β=0.28, 95%CI:0.18-0.38).

**Conclusions:**

Our study suggests the hypothetical role of depression and anxiety as underlying mechanisms in the association between ever having ADHD and obesity in adolescents. When treating children with ADHD, clinicians need to be particularly attentive to whether they show emotional problems and use interventions to eliminate anxiety/depression to protect against obesity.

**Supplementary Information:**

The online version contains supplementary material available at 10.1186/s12888-023-04882-x.

## Introduction

Obesity has become one of the most important public health problems in the United States and many other countries, affecting ~18% of US children and adolescents [1]. The prevalence of pediatric overweight and obesity in all racial and ethnic groups has continued to increase in recent decades [2, 3]. As the prevalence of obesity increases, so does the prevalence of associated comorbidities that affect cardiovascular, endocrine, gastrointestinal, psychosocial and pulmonary health, etc., namely noncommunicable diseases(NCDs) [4]. The 2030 Agenda for Sustainable Development proposed a suite of Sustainable Development Goals (SDGs),including reducing premature mortality from NCDs by one-third and ending ‘all forms of malnutrition’, including obesity(SDG 2.2) [5]. Thus, considering the high prevalence and severe consequences of obesity, it is vital to reveal its influential factors.

Previous studies have suggested that attention deficit/hyperactivity disorder(ADHD) is a risk factor for obesity in both children and adolescents [6-8]. A longitudinal study of 6934 children aged 7-8 years old who were followed up until 16 years old showed that teacher reported ADHD symptoms in childhood could significantly predict obesity in adolescence [7]. Another study of 336 children with ADHD and 665 controls revealed that during the follow-up, children with ADHD were 1.23 (95% CI, 1.00–1.50) times more likely to be obese [9]. However, on the contrary, some studies showed evidence indicating a positive causal effect of body mass index (BMI)on ADHD, rather than the opposite [10]. Thus, given the two contrary views about the influence of ADHD on obesity, it is necessary to further explore the pathway between ADHD and obesity, and the pathway may be complicated and elusive.

Emotional problems have been found to be strongly linked to both ADHD and obesity. A longitudinal genetically sensitive study launched out by Adi Stern et al. provided evidence that ADHD symptoms predicted emotional symptoms, including anxiety/depression, from childhood up to young adulthood through shared genetic influences [11]. Studies have also supported that anxiety in childhood was associated with subsequent obesity after controlling for covariant factors. For example, Claudia et al., in their examination characterizing the impact of anxiety on the severity of obesity among adolescents, showed that anxiety was significantly associated with greater BMI [12]. Youth who met the criteria for a depressive/anxiety disorder were almost 2.5 times as likely to be obese [13]. Moreover,a large cohort study that consistently measured BMI and internalizing symptoms showed that BMI and internalizing symptoms were associated between 7 and 14 years of age [14]. However, to date, few studies have investigated the full model of the complex relationship among ADHD, emotional problems and obesity, especially in adolescents.

Several factors have been suggested to influence the association between ADHD and obesity. The link may be explained by genetic overlap (such as the shared risk allels rs206936 and rs6497416), the underdevelopment of the prefrontal cortex, and behaviors that lead to weight gain and interactions with environmental factors [15-17], such as binge eating behavior, less physical activity, and sleep disruption. Furthermore, recent studies have indicated that the link between ADHD symptoms and excessive body weight could be explained by cumulative psychosocial risks, mainly focusing on family-related risk factors, including over-crowded living conditions and parental education level, etc [9]. Fewer studies have focused on individual risk factors, such as psychological distress.

**Fig. 1:**
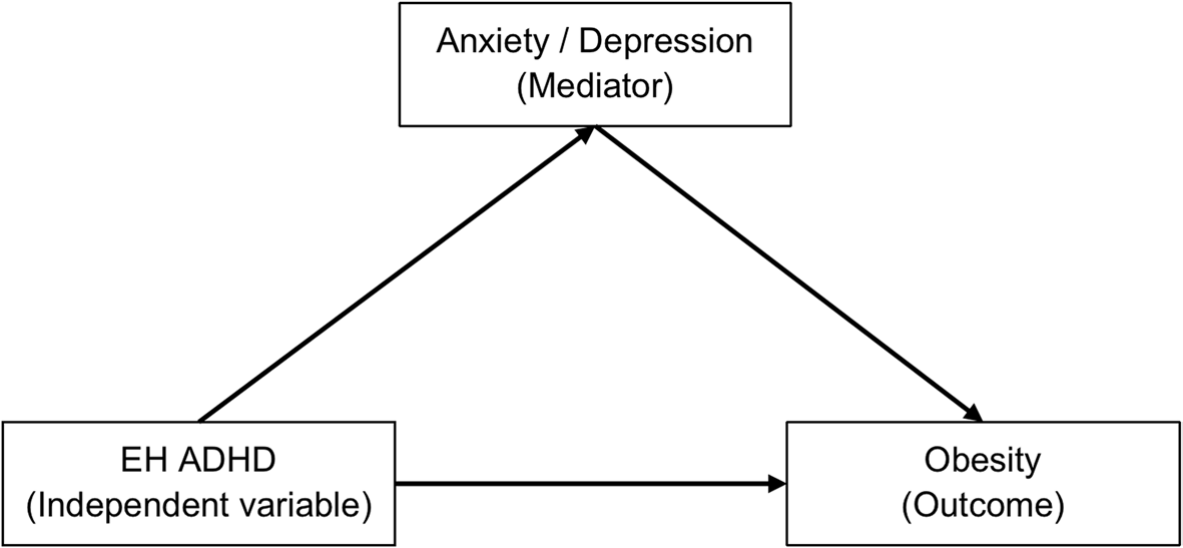
The proposed mediation model. Note: EH ADHD=ever having attention-deficit/hyperactivity disorder

Therefore, this study intended to further examine the relationship among an earlier diagnosis of ADHD, emotional problems and obesity in adolescents by adopting a comprehensive model. It was hypothesized that emotional problems would mediate the relationship between an earlier diagnosis of ADHD and obesity (Fig. 1, The proposed mediation model).

## Methods

### Participants and study design

The National Health Interview Survey (NHIS) is a principal source of information on the health of the civilian noninstitutionalized population of the United States and is publicly available (https://www.cdc.gov/nchs/nhis/nhis_listserv.htm). It is an annual, cross-sectional, in-person household interview survey. Since 1957, the National Center for Health Statistics has been collecting annual data on a broad range of health topics. Data including basic health information, the sociodemographic information of all household members and more extensive information on one sample adult and one sample child per family, are collected by trained personnel from the U.S. Census Bureau through personal face-to-face interviews conducted throughout the year. A multistage sampling technique is used to obtain a representative sample of the U.S. population from each state and the District of Columbia. One child who is represented by a proxy respondent, typically their parent, is randomly selected from each family by the NHIS. Details on the NHIS sample design are published elsewhere [18, 19].

**Fig. 2:**
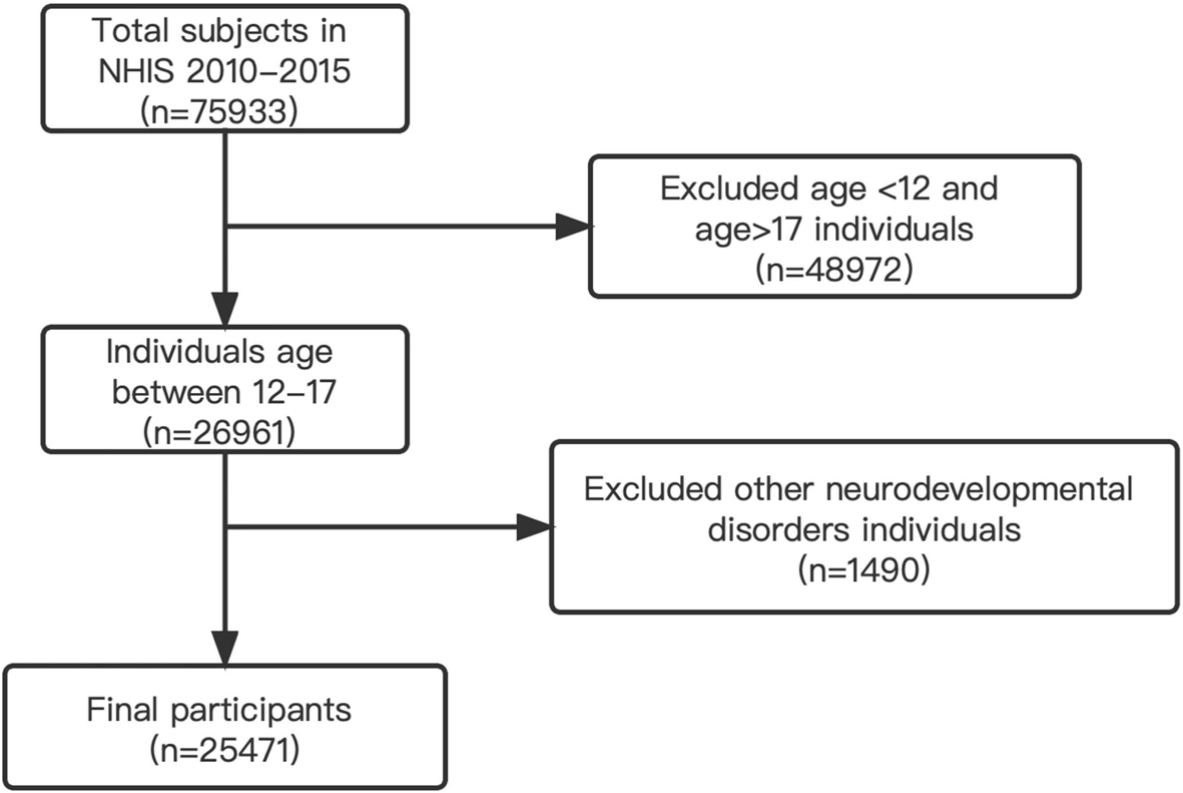
The participant inclusion flowchart

From a total sample of 75,933 participants who were interviewed for the NHIS between 2010 and 2015, a subsample of 26,961 participants (aged 12-17 years) was eligible for this study. After excluding individuals who had ever been told they had mental retardation, autism, Asperger's syndrome, pervasive developmental or autism spectrum disorders, and other relative limitations due to developmental delay, 25,471 adolescents representing a total weighted population of 23,321,066 were included (Fig. 2, The participant inclusion flowchart). We tested our hypothesis by analyzing the data from 2010-1015. Then, the results were verified through analysis of the data from 2016-2018(9,637 adolescents representing 23,229,663 weighted population).

### Measures

#### Obesity

Obesity was defined as a BMI[weight (kilograms)/height (meters) [2]] at or above the 95th percentile for children and teens of the same age and sex, which was calculated from the information that the respondents supplied in response to survey questions regarding height and weight, according to the National Center for Health Statistics Data Table of BMI-for-age Charts (https://www.cdc.gov/growthcharts/html_charts/bmiagerev.htm) [20].

#### Ever having ADHD

Whether participants ever had ADHD was ascertained based on whether the parent answered “yes” or “no” to the question “Has a doctor or health professional ever told you that your child has Attention Deficit Hyperactivity Disorder (ADHD) or Attention Deficit Disorder (ADD)?”

#### Anxiety/depression

Anxiety and depression were defined using separate items derived from the short Strengths and Difficulties Questionnaire (SDQ), which asked parents to report whether their child had many worries or often seemed worried, unhappy, depressed or tearful during the past 6 months. A 3-point Likert scale was used to assess compliance with these items, ranging from 0 (not true) to 2 (certainly true). Responses were dichotomized as positive ("somewhat true" or "certainly true") versus negative ("not true") [21]. There is a high correlation between an SDQ positive screen and a diagnosis made by a clinician [22, 23].

### Statistical analyses

Data analyses were executed with the following steps.

First, to present the characteristics of the respondents, descriptive analyses were performed, expressed as weighted frequencies and percentages.

Second, to assess the association among ever having ADHD, anxiety /depression and obesity, multivariate binary logistic regression models were conducted, with dichotomized outcomes (i.e., ever having ADHD, anxiety, depression) as independent measures and obesity as the dependent measures and adjustment for sex, age, race/ethnicity (Hispanic, Non-Hispanic White, Non-Hispanic Black, Non-Hispanic Asian, Non-Hispanic All other race groups), parental education level (less than a high school diploma, high school diploma or General Educational Development (GED),more than high school), family income ($0-$34999, $35000-$74999, $75000-$99999, $100000 and over) and birth weight, based on their known association with obesity from previous studies [24-29].

Finally, to test the mediation effect of emotional problems (i.e., anxiety, depression) on the relationship between ever having ADHD and obesity, we conducted a follow-up analysis using multivariate binary logistic regression adjusting for sex, age, race/ethnicity, parental education level, family income and birth weight, according to the steps to test for in the mediation analysis of categorical variables [30].

Except for the 95% CI of the mediation effect obtained by the “R Mediation” package of R Statistical Software (R version 4.2.2) [31], all statistical analyses were conducted using the SPSS Statistics for Windows, Version 26.0 Complex Samples module with weighting according to guidelines published by the NHIS [18, 19]. Missing values were excluded from the analysis (pairwise exclusion).

## Results

### Sample characteristics and preliminary analyses

The characteristics of the sample and weighted percent of the population from the 2010-2015 National Health Interview Survey are shown in Table 1. Among the 25,471 adolescents, 13,034 (50.3%) were male. The mean age was 14.50 years (standard deviation [SD] = 0.01), and the mean birth weight was 3318.87 grams (SD = 5.70). Overall, 14.6% of the participants (95% CI 14.0%-15.1%) were classified as being obese. A total of 13% of the participants screened positive for depression, 27.5% screened positive for anxiety, and 10.2% screened positive for ever having ADHD.

**Table 1:** Sample characteristics and weighted percent of the population, 2010-2015 National Health Interview Survey

Variable and level	Estimate	SE	Weighted percent of population (95% CI)	Unweighted count
Obesity	3181314.67	71911.49	14.6 (14.0-15.1)	3776
EH ADHD	2383146.33	61898.40	10.2 (9.8-10.7)	2441
Anxiety	6336497.83	121875.60	27.5 (26.8-28.3)	6777
Depression	2996217.50	74891.11	13.0 (12.5-13.6)	3307
Male sex	11725788.33	178644.01	50.3 (49.5-51.1)	13034
Age (in years)				
12	3913582.67	94410.34	16.8 (16.1-17.4)	3860
13	3932329.67	84767.85	16.9 (16.3-17.4)	3971
14	3750877.17	84265.61	16.1 (15.5-16.7)	4038
15	3931170.50	85705.16	16.9 (16.3-17.5)	4336
16	3977826.17	92170.16	17.1 (16.4-17.7)	4646
17	3815279.67	76151.94	16.4 (15.8-16.9)	4620
Race/ethnicity				
Hispanic	5222906.83	129766.69	22.4 (21.4-23.4)	7278
Non-Hispanic White	13196298.83	237627.06	56.6 (55.4-57.8)	11973
Non-Hispanic Black	3523136.17	93081.43	15.1 (14.4-15.9)	4286
Non-Hispanic Asian	1134880.17	45792.44	4.9 (4.5-5.2)	1635
Non-Hispanic All other race groups	243843.83	31139.54	1.0 (0.8-1.3)	299
Family income				
$0-$34,999	6213081.50	127099.88	28.7 (27.7-29.7)	7590
$35,000-$74,999	6540628.00	124197.31	30.2 (29.3-31.0)	7350
$75,000-$99,999	2839447.83	81795.18	13.1 (12.5-13.7)	2969
$100,000 and over	6080036.33	152821.46	28.1 (27.0-29.2)	5764
Parental education				
Less than a high school diploma	2691009.17	78533.85	12.0 (11.4-12.7)	3441
High school diploma or GED	4284687.50	94281.36	19.2 (18.4-19.9)	4980
More than high school	15392506.17	241753.47	68.8 (67.8-69.8)	15775

*EH ADHD* Ever having attention-deficit/hyperactivity disorder, *SE* Standard Error, *CI* Confidence interval

Table 2 presents the results of three multivariate binary logistic regression models run separately while controlling for sex, race/ethnicity, family income, parental education level, age and birth weight, testing the association among between ever having ADHD, anxiety, depression and obesity. Ever having ADHD was significantly associated with higher odds of obesity, anxiety, and depression. Adolescents with ever having ADHD had statistically higher odds of obesity (AOR 1.33, 95% CI 1.14-1.56), anxiety (AOR 1.36, 95% CI 1.23-1.50), and depression (AOR 1.37, 95% CI 1.18-1.59). Similar results were found when performing data analysis of the 2016-2018 NHIS combined survey in terms of ever having ADHD and depression (Supplementary Table 2s).

**Table 2:** Results of logistic regression analysis predicting odds of obesity, 2010-2015 National Health Interview Survey

Predictor variable and level	Outcome variable: Obesity					
	Model 1^a^ (*n=*19386)		Model 2^b^ (*n=*19229)		Model 3^c^(*n=*19227)	
	AOR	95%CI	AOR	95%CI	AOR	95%CI
EH ADHD						
Yes	1.33***	1.14-1.56				
No	Reference					
Anxiety						
Yes			1.36***	1.23-1.50		
No			Reference			
Depression						
Yes					1.37***	1.18-1.59
No					Reference	
Sex						
Male	1.42***	1.28-1.58	1.49***	1.35-1.65	1.48***	1.33-1.64
Female	Reference		Reference		Reference	
Race/ethnicity						
Hispanic	1.05	0.61-1.81	1.06	0.62-1.81	1.05	0.61-1.80
Non-Hispanic White	0.73	0.43-1.26	0.75	0.44-1.28	0.75	0.44-1.29
Non-Hispanic Black	1.11	0.65-1.90	1.16	0.68-1.97	1.14	0.67-1.95
Non-Hispanic Asian	0.52*	0.28-0.94	0.53*	0.29-0.96	0.52*	0.28-0.95
Non-Hispanic All other race groups	Reference		Reference		Reference	
Family income						
$0-$34,999	2.15***	1.81-2.56	2.13***	1.78-2.54	2.12***	1.78-2.53
$35,000-$74,999	1.87***	1.59-2.20	1.85***	1.57-2.17	1.85***	1.57-2.17
$75,000-$99,999	1.61***	1.31-1.97	1.57***	1.28-1.93	1.58***	1.28-1.94
$100,000 and over	Reference		Reference		Reference	
Parental education						
Less than a high school diploma	1.34**	1.13-1.58	1.36**	1.14-1.61	1.33**	1.12-1.58
High school diploma or GED	1.23**	1.07-1.40	1.25**	1.09-1.43	1.24**	1.08-1.42
More than high school	Reference		Reference		Reference	
Age (in years)	0.92***	0.90-0.95	0.92***	0.89-0.95	0.92***	0.89-0.95
Birth weight	1.00***	1.00-1.00	1.00***	1.00-1.00	1.00***	1.00-1.00

*EH ADHD* Ever having attention-deficit/hyperactivity disorder, *AOR* Adjusted odds ratio, *CI* Confidence interval

^*^P<0.05, **P<0.01, ***P<0.001

^a^Missing data, *n =* 6085

^b^Missing data, *n =* 6242

^c^Missing data, *n =* 5244

### Testing for mediation effect

Table 3 shows the results of two multiple logistic regression models run separately while controlling for covariates to test the mediating role of anxiety on the relationship between ever having ADHD and obesity. Model 1 showed that ever having ADHD significantly predicted the odds of anxiety (AOR 2.43, 95% CI 2.12-2.77). Model 2 showed that anxiety significantly predicted the odds of obesity (AOR 1.32, 95% CI 1.20-1.47), after adjusting for ever having ADHD. The indirect effect of ever having ADHD on obesity via anxiety was significant (indirect effect = 0.25, 95% CI 0.15-0.35), as determined by the “RMediation” package of R Statistical Software. However, analysis of the 2016-2018 NHIS combined survey data did not produce similar results (Supplementary Table 3s.a).

**Table 3:** Results of logistic regression analysis testing the mediating role of anxiety on the relation between ever having ADHD and obesity, 2010-2015 National Health Interview Survey

Predictors	Model 1^a^ (Anxiety,*n=*19971)		Model 2^b^ (Obesity, *n=*19221)	
	β (SE)	AOR (95%CI)	β (SE)	AOR (95%CI)
EH ADHD				
Yes	0.89 (0.07)***	2.43 (2.12-2.77)	0.24 (0.08)**	1.27 (1.08-1.49)
No	Reference		Reference	
Anxiety				
Yes			0.28 (0.05)***	1.32 (1.20-1.47)
No			Reference	
Nagelkerke R2	0.05		0.06	

AORs are adjusted for sex, race/ethnicity, family income, parental education, age, birth weight. Each column is a logistic regression on model that predicts the criterion at the top of the column

*EH ADHD* Ever having attention-deficit/hyperactivity disorder, *AOR* Adjusted odds ratio, *CI* Confidence interval

^*^P<0.05, **P<0.01, ***P<0.001

^a^Missing data, *n =* 5500

^b^Missing data, *n =* 6250

Table 4 shows the results of two multiple logistic regression models run separately while controlling for sex, race/ethnicity, family income, parental education, age and birth weight to test the mediating role of depression on the relation between ever having ADHD and obesity. Model 1 showed that ever having ADHD significantly predicted the odds of depression (AOR 3.00, 95% CI 2.58-3.49). Model 2 showed that depression significantly predicted the odds of obesity (AOR 1.32, 95% CI 1.14-1.53), adjusted for ever having ADHD. The indirect effect of ever having ADHD on obesity via depression was significant (indirect effect = 0.31, 95% CI 0.14-0.48), as determined by the “RMediation” package of R Statistical Software. Similar results were found when performing the analysis for the data from the 2016-2018 NHIS combined survey (Supplementary Table 3s.b).

**Table 4:** Results of logistic regression analysis testing the mediating role of depression on the relation between ever having ADHD and obesity, 2010-2015 National Health Interview Survey

Predictors	Model 1^a^ (Depression,*n=*19970)		Model 2^b^ (Obesity, *n=*19219)	
	β (SE)	AOR (95%CI)	β (SE)	AOR (95%CI)
EH ADHD				
Yes	1.10 (0.08)***	3.00 (2.58-3.49)	0.25 (0.08)**	1.28 (1.10-1.50)
No	Reference		Reference	
Depression				
Yes			0.28 (0.08)***	1.32 (1.14-1.53)
No			Reference	
Nagelkerke R2	0.07		0.06	

AORs are adjusted for sex, race/ethnicity, family income, parental education, age, birth weight. Each column is a logistic regression on model that predicts the criterion at the top of the column

*EH ADHD* Ever having attention-deficit/hyperactivity disorder, *AOR* Adjusted odds ratio, *CI* Confidence interval

^*^P<0.05, **P<0.01,***P<0.001

^a^Missing data, *n =* 5501

^b^Missing data, *n =* 6252

## Discussion

This study included a large cross-sectional sample of adolescents and formulated a mediated model to investigate the influence of ever having ADHD and emotional problems on adolescent obesity. The results provided support for the hypothesis that ever having ADHD was indirectly associated with adolescent obesity through anxiety/depression. These results contribute to a better understanding of the influential factors in the development of obesity in individuals with ADHD, which may be helpful for improving health in this group of population.

The association between ADHD and obesity is now well established in adults, while whether it is the same in children and adolescents remains unclear [32-35]. In the current study, we found a direct link between ever having ADHD and adolescent obesity, and further analysis showed that depression and anxiety partially explained the association between the two conditions. There are limited data on the association between ever having ADHD and adolescent obesity. Donnchadha et al. used a longitudinal sample, and logistic regression indicated that ADHD status was not associated with overweight/obesity at 9 or 13 years of age, but children with ADHD at 9 years of age were significantly more likely to be overweight/obese at 13 years of age than those without ADHD, and this relationship was largely explained by a variety of psychosocial factors [36]. Previously,, Daphne et al. conducted a prospective longitudinal study consisting of 3294 community participants aged from 4-16 years old and found that ADHD in adolescence was not associated with increased adult BMI, while childhood hyperactivity seemed to be associated with adult BMI [37]. Moreover, this association was completely accounted for by conduct disturbance. A longitudinal population-based study conducted by Roxana et al. consisting of a birth cohort indicated that childhood ADHD was associated with obesity during childhood and young adulthood only in females [9]. In line with this finding, Thais et al. recently utilized a population-based birth cohort and discovered that ADHD at 11 years of age predicted a higher BMI at 15 years of age [38]. However, Bezawit et al. found that the link between ADHD and BMI was stable from late childhood (10-12 years) up to early adulthood (20-22 years),while no longitudinal direct effects were found between ADHD symptoms and BMI, suggesting that potential causal effects may be established earlier in childhood [39]. Recently, Nora et al. showed that the presence of anxiety and ADHD in adolescents increased the odds of obesity, with children with ADHD having an almost 50% increased odds of obesity [23]. Moreover, cross-sectional studies have also supported that the mean age of onset of ADHD preceded the mean age of onset of obesity [7, 37, 40, 41].

A study by Pauli-Pott et al. concluded that the relationship between ADHD symptoms and excessive body weight was accounted for by psychosocial cumulative risks associated with the family environment, including parental education level, parental separation and overcrowed living conditions [9]. Based on this, the current study provided further clarification regarding the possible mechanisms by which ever having ADHD may lead to adolescent obesity at the individual risk factor level. Our study identified anxiety/depression as mediators of the relationship after controlling for family environmental risk factors. To our knowledge, this study was the first to examine the mediating role of emotional problems in the association of ADHD and obesity in adolescents.

The mechanisms underlying the mediating role of depression or anxiety in the relationship between ADHD and obesity are not well understood. Current evidence suggests the hypothesis of the “psychopathology-to-obesity” pathway.First, shared genetic vulnerability. Recent studies have revealed several pleiotropic genes that might be shared between mood disorders and obesity, such as FTO (encoding fatmass and obesity-associated protein), BDNF (encoding brain-derived neurotrophic factor), POMC (encoding proopiomelanocortin), and IGF1 (encoding insulin-like growth factor 1), as well as shared genetic pathways involving serotonin and dopamine receptor signaling, leptin signaling, circadian rhythm signaling, axonal guidance signaling, and corticotropin-releasing hormone signaling [34]. These are the molecular genetic basis for constructing the hypothesis of the “psychopathology-to-obesity” pathway.

Second, in terms of brain structure and function, ADHD, emotional problems and obesity have been hypothesized to share common underlying abnormalities in brain reward sections, emotion regulation processes and executive functions [42]. Regarding the reward system, recent neuroimaging studies have shown that striatal reactivity in the monetary incentive delay partially mediates the genetic predisposition for ADHD and BMI [43]. Reward network studies did not show a consistent overlap between ADHD and depression, so prospective longitudinal studies are needed in the future [44]. In terms of emotion regulation processes, which are commonly seen both in ADHD and emotional disorders, mood lability and poor emotion-regulatory capabilities predispose these populations to abnormal eating behaviors, such as binge eating [42]. Executive dysfunction, which is the core characteristic of ADHD, especially poor cortical inhibitory control and impulsivity, could lead to overeating and the development of food addiction. Moreover, recent studies have shown associations between emotional disorders and various levels of dysexecutive features, and evidence has showed that motivational-emotional (“hot executive”) dysfunction underlies a subset of nonnormative eating behaviors [8].

Third,the psycho-immune-neuro-endocrine network. Accumulating evidence suggests that patients with neurodevelopmental disorders share inherent features of dysregulated homeostasis systems, including the hypothalamic‒pituitary‒adrenal (HPA)-axis, leptin-ghrelin system, insulin resistance, dysbiosis, endocrine and autonomic dysfunctions, and the inflammatory response, which ultimately results in excess adiposity and dysmetabolism [45]. In addition, a short sleep duration, a lack of physical exercise, and medicine use might also mediate the association between neurodevelopmental disorders and obesity [34].

In addition, obesity and emotional problems share common sociodemographic risk factors, including sex, birth weight, and parental education level, etc [46-48]. Based on this, we set these factors set as control variables in this study.

Specific to depression, our mediation analysis further revealed that depression mediated 27.87% of the risk effect of ADHD on obesity. To date, few studies have explored the mediating role of depression in the relationship between ADHD and obesity. In 2009, a cross-sectional study in adults showed that binge eating disorder, but not depression, partially mediated the associations between ADHD and obesity [49]. However, no corresponding study in children has been reported. In 2014, a study involving 171 adult women indicated that depressive symptoms, followed by ADHD inattention symptoms and impulsivity, could well predict the presence of binge eating in obese individuals [50]. Furthermore, a randomized controlled trial (RCT) has already been performed to establish the efficacy of nonpharmacological interventions targeting the prevention of comorbid depression and obesity among adolescents and young adults with ADHD [51].

A major strength of the NHIS is the ability to generalize findings to populations. Furthermore, using the 2016-2018 data, we found more supporting evidence that ever having ADHD, depression or anxiety increased the risk of obesity in adolescents, as well as the mediating role of depression in the association between ever having ADHD and obesity. Several limitations of this study should be noted. First, we obtained the diagnosis of ADHD/ADD through retrospective parent interviews, which might be subject to recall bias, and we could not differentiate ADHD subtypes. Second, the participating children’s heights and weights were reported by their parents rather than clinically measured. Third, we did not take into account other variables that may affect body weight, such as diet, lifestyle, physical activity, and sleep conditions. Fourth, it is possible that ADHD medications may have weight‐related side effects, which we did not consider in this study.

In conclusion, increasing evidence suggests a connection between ADHD and obesity in both children and adolescents. This understanding may strengthen the prevention and treatment of obesity. It is worth noting that recognizing the susceptible factors and populations of obesity can improve the effectiveness of obesity treatment. Based on this, our study indicates that ever having ADHD is associated with an increased risk of obesity in adolescents, and this association is partially mediated by emotional problems (depression/anxiety). The results suggest that emotional problems might be effective areas for interventions aimed at reducing obesity risk in adolescents with ADHD/ADD.

## Supplementary Information

**Additional file 1:** **Table 1s.** Sample characteristics and weighted percent of the population, 2016-2018 National Health Interview Survey. **Table 2s.** Results of logistic regression analysis predicting odds of obesity, 2016-2018 National Health Interview Survey. **Table 3s.a. **Results of logistic regression analysis testing the mediating role of anxiety on the relation between ever having ADHD and obesity, 2016-2018 National Health Interview Survey. **Table 3s.b.** Results of logistic regression analysis testing the mediating role of depression on the relation between ever having ADHD and obesity, 2016-2018 National Health Interview Survey.

## Data Availability

The datasets generated and/or analyzed during the current study are available at https://www.cdc.gov/nchs/nhis/nhis_listserv.htm.
