# Supplementary material for: Emotional problems mediate the association between attention deficit/hyperactivity disorder and obesity in adolescents
Source: BMC Psychiatry. 2023 May 31;23:381. doi: 10.1186/s12888-023-04882-x (PMC10230792; doi:10.1186/s12888-023-04882-x)
Supplement: Supplementary file 1 — Additional file 1: Table 1s. Sample characteristics and weighted percent of the population, 2016-2018 National Health Interview Survey. Table 2s. Results of logistic regression analysis predicting odds of obesity, 2016-2018 National Health Interview Survey. Table 3s.a. Results of logistic regression analysis testing the mediating role of anxiety on the relation between ever having ADHD and obesity, 2016-2018 National Health Interview Survey. Table 3s.b. Results of logistic regression analysis testing the mediating role of depression on the relation between ever having ADHD and obesity, 2016-2018 National Health Interview Survey. [file 12888_2023_4882_MOESM1_ESM.docx]

| Table 1s | | | | |
| --- | --- | --- | --- | --- |
| Sample characteristics and weighted percent of the population, 2016-2018 National Health Interview Survey | | | | |
| Variable and level | Estimate | SE | Weighted percent of population (95% CI) | Unweighted count |
| Obesity | 3132180.67 | 126822.15 | 14.6 (13.6-15.6) | 1363 |
| EH ADHD | 2625129.67 | 108271.08 | 11.3 (10.5-12.1) | 1145 |
| Anxiety | 6557000.67 | 203759.20 | 28.5 (27.3-29.8) | 2905 |
| Depression | 3140775.00 | 118170.04 | 13.7 (12.8-14.5) | 1383 |
| Male sex | 11460418.33 | 325788.13 | 49.3 (48.1-50.6) | 4849 |
| Age (in years) |  |  |  |  |
| 12 | 3881678.00 | 158374.15 | 16.7 (15.7-17.8) | 1461 |
| 13 | 3850542.67 | 145546.75 | 16.6 (15.6-17.6) | 1488 |
| 14 | 3842976.67 | 157026.00 | 16.5 (15.6-17.6) | 1511 |
| 15 | 3929200.33 | 146311.19 | 16.9 (16.0-17.8) | 1649 |
| 16 | 4043303.00 | 154043.81 | 17.4 (16.5-18.3) | 1795 |
| 17 | 3681961.67 | 138734.19 | 15.9 (15.0-16.8) | 1733 |
| Race/ethnicity |  |  |  |  |
| Hispanic | 5675302.00 | 289966.34 | 24.4 (22.6-26.4) | 2166 |
| Non-Hispanic White | 12545350.00 | 364061.54 | 54.0 (51.9-56.1) | 5468 |
| Non-Hispanic Black | 3349289.00 | 168653.60 | 14.4 (13.2-15.7) | 1229 |
| Non-Hispanic Asian | 1344765.00 | 97861.20 | 5.8 (5.1-6.6) | 604 |
| Non-Hispanic All other race groups | 314956.33 | 69188.76 | 1.4 (0.9-2.1) | 170 |
| Family income |  |  |  |  |
| $0-$34,999 | 4883261.00 | 186860.24 | 23.5 (22.1-24.9) | 1989 |
| $35,000-$74,999 | 5686380.33 | 204224.12 | 27.3 (26.0-28.7) | 2331 |
| $75,000-$99,999 | 2446684.67 | 106027.02 | 11.8 (10.9-12.6) | 1072 |
| $100,000 and over | 7788750.67 | 280956.06 | 37.4 (35.7-39.2) | 3287 |
| Parental education |  |  |  |  |
| Less than a high school diploma | 2589051.33 | 159383.75 | 11.6 (10.5-12.8) | 898 |
| High school diploma or GED | 3704644.00 | 141188.05 | 16.6 (15.6-17.6) | 1583 |
| More than high school | 16041482.33 | 440485.75 | 71.8 (70.3-73.3) | 6721 |
| Note. EH ADHD=Ever having attention-deficit/hyperactivity disorder; SE=Standard Error; CI=confidence interval. | | | | |

| Table 2s |  |  |  |  |  |  |  |  |
| --- | --- | --- | --- | --- | --- | --- | --- | --- |
| Results of logistic regression analysis predicting odds of obesity, 2016-2018 National Health Interview Survey | | | | | | | | |
| Predictor variable and level | Outcome variable: Obesity | | | | | | | |
|  | Model 1† (n=7159) | |  | Model 2‡ (n=7115) | |  | Model 3§(n=7114) | |
|  | AOR | 95%CI |  | AOR | 95%CI |  | AOR | 95%CI |
| EH ADHD |  |  |  |  |  |  |  |  |
| Yes | 1.35* | 1.06-1.71 |  |  |  |  |  |  |
| No | Reference |  |  |  |  |  |  |  |
| Anxiety |  |  |  |  |  |  |  |  |
| Yes |  |  |  | 1.19 | 0.99-1.42 |  |  |  |
| No |  |  |  | Reference |  |  |  |  |
| Depression |  |  |  |  |  |  |  |  |
| Yes |  |  |  |  |  |  | 1.34* | 1.07-1.69 |
| No |  |  |  |  |  |  | Reference |  |
| Sex |  |  |  |  |  |  |  |  |
| Male | 1.61*** | 1.36-1.89 |  | 1.67*** | 1.41-1.96 |  | 1.67*** | 1.42-1.97 |
| Female | Reference |  |  | Reference |  |  | Reference |  |
| Race/ethnicity |  |  |  |  |  |  |  |  |
| Hispanic | 0.47* | 0.26-0.84 |  | 0.45** | 0.25-0.81 |  | 0.46** | 0.25-0.82 |
| Non-Hispanic White | 0.40** | 0.23-0.69 |  | 0.39** | 0.22-0.69 |  | 0.40** | 0.23-0.69 |
| Non-Hispanic Black | 0.50* | 0.28-0.89 |  | 0.51* | 0.29-0.92 |  | 0.52* | 0.29-0.92 |
| Non-Hispanic Asian | 0.24*** | 0.12-0.48 |  | 0.23*** | 0.12-0.46 |  | 0.23*** | 0.11-0.46 |
| Non-Hispanic All other race groups | Reference |  |  | Reference |  |  | Reference |  |
| Family income |  |  |  |  |  |  |  |  |
| $0-$34,999 | 2.06*** | 1.62-2.61 |  | 2.08*** | 1.64-2.64 |  | 2.04*** | 1.60-2.60 |
| $35,000-$74,999 | 2.04*** | 1.63-2.56 |  | 2.05*** | 1.63-2.56 |  | 2.04*** | 1.62-2.56 |
| $75,000-$99,999 | 1.68*** | 1.28-2.20 |  | 1.65*** | 1.26-2.16 |  | 1.65*** | 1.26-2.17 |
| $100,000 and over | Reference |  |  | Reference |  |  | Reference |  |
| Parental education |  |  |  |  |  |  |  |  |
| Less than a high school diploma | 1.41* | 1.06-1.88 |  | 1.47** | 1.11-1.96 |  | 1.45* | 1.09-1.93 |
| High school diploma or GED | 1.30* | 1.06-1.58 |  | 1.32** | 1.08-1.62 |  | 1.31** | 1.08-1.60 |
| More than high school | Reference |  |  | Reference |  |  | Reference |  |
| Age (in years) | 0.99 | 0.95-1.04 |  | 0.99 | 0.94-1.04 |  | 0.99 | 0.95-1.04 |
| Birth weight | 1.00 | 1.00-1.00 |  | 1.00 | 1.00-1.00 |  | 1.00 | 1.00-1.00 |
| Note. EH ADHD=Ever having attention-deficit/hyperactivity disorder; AOR=adjusted odds ratio; CI=confidence interval；*P＜0.05, **P＜0.01,***P＜0.001. † Missing data, n = 2478 ‡ Missing data, n = 2522 § Missing data, n = 2523 | | | | | | | | |

| Table 3s.a |  |  |  |  |  |
| --- | --- | --- | --- | --- | --- |
| Results of logistic regression analysis testing the mediating role of anxiety on the relation between ever having ADHD and obesity, 2016-2018 National Health Interview Survey | | | | | |
| Predictors | Model 1† (Anxiety,n=7381) | |  | Model 2§ (Obesity, n=7112) | |
|  | β (SE) | AOR (95%CI) |  | β (SE) | AOR (95%CI) |
| EH ADHD |  |  |  |  |  |
| Yes | 0.68 (0.09)*** | 1.97 (1.64-2.35) |  | 0.28 (0.12)* | 1.33 (1.05-1.69) |
| No | Reference |  |  | Reference |  |
| Anxiety |  |  |  |  |  |
| Yes |  |  |  | 0.15 (0.09) | 1.16 (0.97-1.39) |
| No |  |  |  | Reference |  |
|  |  |  |  |  |  |
| Nagelkerke R2 | 0.07 | |  | 0.07 | |
| Note. AORs are adjusted for sex, race/ethnicity, family income, parental education, age, birth weight. Each column is a logistic regression on model that predicts the criterion at the top of the column. EH ADHD=Ever having attention-deficit/hyperactivity disorder; AOR=adjusted odds ratio; CI=confidence interval；*P＜0.05, **P＜0.01,***P＜0.001. † Missing data, n = 2256 § Missing data, n = 2525 | | | | | |

| Table 3s.b |  |  |  |  |  |
| --- | --- | --- | --- | --- | --- |
| Results of logistic regression analysis testing the mediating role of depression on the relation between ever having ADHD and obesity, 2016-2018 National Health Interview Survey | | | | | |
| Predictors | Model 1‡ (Depression,n=7378) | |  | Model 2§ (Obesity, n=7110) | |
|  | β (SE) | AOR (95%CI) |  | β (SE) | AOR (95%CI) |
| EH ADHD |  |  |  |  |  |
| Yes | 0.89 (0.10)*** | 2.43 (1.98-2.99) |  | 0.27 (0.12)* | 1.31 (1.03-1.67) |
| No | Reference |  |  | Reference |  |
| Depression |  |  |  |  |  |
| Yes |  |  |  | 0.26 (0.12)* | 1.30 (1.03-1.63) |
| No |  |  |  | Reference |  |
|  |  |  |  |  |  |
| Nagelkerke R2 | 0.06 | |  | 0.07 | |
| Note. AORs are adjusted for sex, race/ethnicity, family income, parental education, age, birth weight. Each column is a logistic regression on model that predicts the criterion at the top of the column. EH ADHD=Ever having attention-deficit/hyperactivity disorder; AOR=adjusted odds ratio; CI=confidence interval；*P＜0.05, **P＜0.01,***P＜0.001. ‡ Missing data, n = 2259 § Missing data, n = 2527 | | | | | |
